# Discovery of Landscape Phage Probes Against Cellular Communication Network Factor 1 (CCN1/Cyr61)

**DOI:** 10.3390/v17091273

**Published:** 2025-09-19

**Authors:** James W. Gillespie, Valery A. Petrenko

**Affiliations:** Department of Pathobiology, College of Veterinary Medicine, Auburn University, Auburn, AL 36849, USA

**Keywords:** Cellular Communication Network Factor 1, CCN1, Cyr61, breast cancer, phage display, diagnostics

## Abstract

Detection of cancer biomarkers at the earliest stages of disease progression is commonly assumed to extend the overall quality of life for cancer patients as the result of earlier clinical management of the disease. Therefore, there is an urgent need for the development of standardized, sensitive, robust, and commonly available screening and diagnostic tools for detecting the earliest signals of neoplastic pathology progression. Recently, a new paradigm of cancer control, known as multi-cancer detection (MCD), evolved, which measures the composition of cancer-related molecular analytes in the patient’s fluids using minimally invasive techniques. In this respect, the “Holy Grail” of cancer researchers and bioengineers for decades has been composing a repertoire or molecular sensing probes that would allow for the diagnosis, prognosis, and monitoring of cancer diseases via their interaction with cell-secreted and cell-associated cancer antigens and biomarkers. Therefore, the current trend in screening and detection of cancer-related pathologies is the development of portable biosensors for mobile laboratories and individual use. Phage display, since its conception by George Smith 40 years ago, has emerged as a premier tool for molecular evolution in molecular biology with widespread applications including identification and screening of cancer biomarkers, such as Circulating Cellular Communication Network Factor 1 (CCN1), an extracellular matrix-associated signaling protein responsible for a variety of cellular functions and has been shown to be overexpressed as part of the response to various pathologies including cancer. We hypothesize that CCN1 protein can be used as a soluble marker for the early detection of breast cancer in a multi-cancer detection (MCD) platform. However, validated probes have not been identified to date. Here, we screened the multi-billion clone landscape phage display library for phages interacting specifically with immobilized CCN1 protein. Through our study, we discovered a panel of 26 different phage-fused peptides interacting selectively with CCN1 protein that can serve for development of a novel phage-based diagnostic platform to monitor changes in CCN1 serum concentration by liquid biopsy.

## 1. Introduction

**Figure 1:**
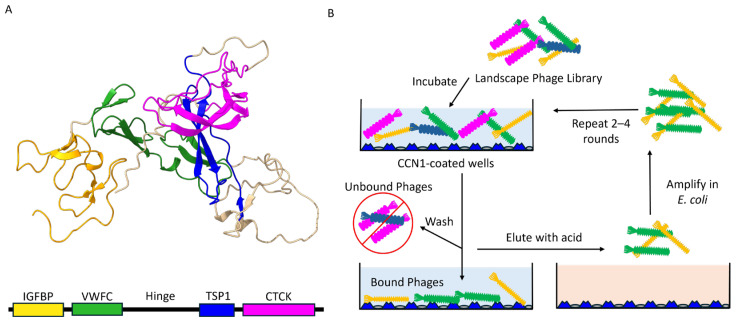
(**A**) 3D protein structure of CCN1 protein predicted by AlphaFold showing four distinct functional domains including: (1) an Insulin-like Growth Factor Binding domain (IGFBP) in yellow, (2) a Von Willebrand Factor Type C domain (VWFC) in green, (3) a Thrombospondin Type-1 domain (TSP1) in blue and (4) a C-terminal Cystine Knot-like domain (CTCK) in magenta. A central hinge region links VWFC and TSP1 domains and is highly susceptible to cleavage by proteases. (**B**) Schematic overview of the phage selection procedure. Briefly, a landscape phage display library is added to CCN1-coated plates (blue triangles) and incubated for 1 h. Unbound and weakly bound phages are washed away and removed. Bound phages are eluted with mild acid treatment, concentrated, and amplified in *E. coli* host cells. The selection process is repeated 2–4 times using amplified phage libraries as input prior to identification and screening of candidate phage clones.

Breast cancer remains a significant global health concern requiring continued development of improved diagnostic and therapeutic strategies to enhance overall patient quality of life. Early-stage detection of malignant phenotypes is critical for improving patient outcomes and increasing overall survival rates. Circulating Cellular Communication Network Factor 1 (CCN1) also known as the Cysteine-rich Angiogenic Inducer 61 (CYR61) protein (Figure 1A) is a secreted, multi-domain, extracellular matrix-associated signaling protein that controls a variety of cellular functions including the regulation of cell proliferation, adhesion, migration, angiogenesis, and apoptosis [1,2]. Aberrant expression of CCN1 is implicated in the pathogenesis and progression of several cancer types. CCN1 exhibits both pro-tumorigenic and tumor-suppressive activities depending on the primary site of malignancy and context within the local microenvironment [3]. Elevated CCN1/CYR61 levels is associated with poor prognosis for various cancers including breast cancer [4,5], prostate cancer [6,7], gastric cancer [8], oral squamous cell carcinoma [9], lung cancer [10,11,12], and hepatocellular carcinoma [13]. CCN1 expression levels correlate with invasiveness and overall aggressiveness in metastatic triple-negative and basal-like breast cancer cell types. However, designing treatment strategies based on breast cancer subtype and CCN1 status have not been thoroughly studied [14]. CCN1 has been suggested as a potential serum biomarker for early detection of cancers with an aggressive phenotype using a liquid biopsy format [14]. Recent trials assessing CCN1 plasma concentrations in breast and lung cancer patients, compared to healthy control populations, have shown elevated expression levels at the earliest stages of breast cancer development [15]. CCN1 expression status can be used to discriminate between malignant verses benign tumor lesions, making it a promising biomarker to use in conjunction with existing diagnostic tools to establish improved patient treatment plans [15].

Molecular probes commonly used to discriminate between analytes in a complex diagnostic sample include antibodies [16], nucleic acid oligomers [17], recombinant proteins, phage-displayed peptides, and enzymes [18]. Antibodies and their various structural formats including fragment antigen binding (Fab) domains, single-chain variable fragment (scFv) domains, nanobodies, chimeric antibodies, and humanized antibodies have been studied extensively to characterize specific protein interactions in a complex sample matrix [19,20]. Antibodies have been a valuable molecular tool for studying protein interactions in a complex sample. However, antibodies have their own limitations and manufacturing challenges which necessitate the development of alternate strategies for designing probes for use in diagnostic assays. Generation of recombinant antibodies based on phage display technologies has been in active development for several decades [21,22]. Traditionally, antibody fragments are displayed at the N-terminus of the mature p3 coat protein of filamentous bacteriophages like M13, fd, or f1. In these type-3 display systems, the displayed protein or antibody fragment is presented as a single copy per phage particle and results in monovalent interactions between the phage and desired target antigen. Recombinant antibodies with high affinity can be identified by screening highly diverse type-3 antibody display libraries against a desired antigen target.

Alternatively, display of short 8 or 9 amino acid peptide fragments fused to the N-terminus of the mature p8 coat protein, represented by ~4000 identical copies, produces phage particles with structurally unique molecular surfaces [23]. These multivalent, type-8 phage particles, or landscape phages, were used as substitute detection probes. Replacing traditional antibody-based detection systems [24,25] for several classes of targets including proteins [26,27], bacterial cells [28], and mammalian cells [29]. The unique molecular architecture of landscape phages allows identification of peptides that create a molecular interface suitable for binding various targets with increased avidity compared to an isolated peptide encoding the same sequence. Whole phage particles can be used to engineer surfaces with a dense array of capture probes in an organized format suitable for development of various diagnostic and biosensor applications (Figure 1B) [30,31]. Phage biodetection systems based on landscape phage probes are more resistant to thermal and chemical degradation compared with traditional antibody detection interfaces, making them ideal for use in harsh or unstable environments [32].

We hypothesized that the structurally unique landscape phage display libraries would be a rich source of peptide ligands that could bind soluble human CCN1 protein and could ultimately be used as capture probes for a variety of diagnostic assay platforms. To test this hypothesis and generate a panel of unique materials for future study, we screened a multibillion-clone landscape phage display library against a purified, recombinant human CCN1 protein in 96-well microplate format and identified a panel of candidate phages that could be used to develop a bacteriophage-based diagnostic assay.

## 2. Materials and Methods

### 2.1. Landscape Phage Display Library

The multibillion-clone f8/9 phage display library was constructed and described in detail previously [33,34]. The f8/9 landscape phage library was shown to have a diversity of ~1.2 × 10^9^ unique clones with enrichment or depletion of specific amino acid residues based on their position within the major coat protein [34]. Phage clones isolated from the f8/9 phage library are identified by the N-terminal fusion peptide displayed on the surface of the phage. Peptide fusions used in this study represent the full-length, 55-mer amino acid sequence of the recombinant, mature p8 protein as follows: NH_2_-A**XXXXXXXXX**PAKAAFDSLQASATEYIGYAWAMVVVIVGATIGIKLFKKFTSGAS-COOH, where X can be any random amino acid based on the library design. All common bacteriophage handling and phage display techniques, including bacteriophage isolation, propagation, titering and sequencing were described and validated previously [35,36].

### 2.2. Selection of CCN1 Binding Phages

A schematic overview of the selection procedure is presented in Figure 1B.

(A) Depletion and First Round Selection—A freshly prepared aliquot of full-length, non-glycosylated, recombinant human CCN1/CYR61 protein (#cyt-164; Prospec, Ness Ziona, Israel) was diluted to 10 µg/mL in 1X Tris-Buffered Saline (TBS), pH 7.5 coating buffer. Eight wells of a high-binding capacity 96-well microplate (#9018; Corning, Corning, NY, USA) were coated with 50 µL of recombinant CCN1 protein (~500 ng/well). An additional eight wells were coated with 50 µL Bovine Serum Albumin (BSA) protein (~500 µg/well) diluted in coating buffer (1X TBS, pH 7.5). Proteins were adsorbed to the wells at 4 °C overnight. Unbound proteins were removed and wells blocked with blocking buffer (1X TBS, pH 7.5/1.0% BSA) at room temperature (20 °C) for 1 h. Wells were thoroughly rinsed three times with 200 µL wash buffer (1X TBS, pH 7.5/0.5% Tween 20).

A 20 µL aliquot of the multibillion-clone f8/9 phage display library containing ~5.5 × 10^11^ virions, with each unique fusion peptide being represented by several hundred virus particles, was diluted in library dilution buffer (1X TBS, pH 7.5/0.5% Tween 20/0.01% BSA) in low-bind microcentrifuge tubes (#3207; Corning, Corning, New York, NY, USA). Prior to the first round of selection, non-desired clones interacting with unrelated targets were removed from the library using two different depletion protocols: (1) depletion against plastic followed by depletion against BSA, and (2) depletion against BSA. For each depletion step, 50 µL of the diluted f8/9 phage library were added to eight wells of a treated 96-well microplate and incubated at room temperature for 1 h. After depletion, the library was transferred to neighboring wells containing adsorbed CCN1 protein and incubated with gentle rocking at room temperature for 1 h. Wells were washed and phages recovered as in Section B below.

(B) Washing and Sublibrary Propagation—Unbound phages were removed from each well and combined into a single 1.7 mL microcentrifuge tube for titering. Each well was washed ten times with 200 µL wash buffer (1X TBS, pH 7.5/0.5% Tween 20) for 5 min before removing and collecting the washes from each well into a single 2.0 mL microcentrifuge tube per wash for titering. Following extensive washing, 100 µL elution buffer (200 mM Glycine-HCl, pH 2.2/1.0 mg/mL BSA) was added to each well and incubated with gentle rocking at room temperature for 10 min to elute bound phage clones. Eluted phages were collected into a single 1.7 mL microcentrifuge tube and neutralized by addition of 120 µL neutralization buffer (1.0 M Tris-HCl, pH 9.1). Eluted phages were concentrated in pre-rinsed 100 kDa MWCO Amicon Ultra-0.5 centrifugal filters (#UFC510008; MilliporeSigma, Burlington, MA, USA) by centrifuging at 14,000× *g* for 20 min at room temperature. Concentrated phages were washed four times by gently rinsing the surface of the filter membrane with 500 µL 1X TBS, pH 7.5 and centrifuging at 14,000× *g* for 20 min until phages were collected in a volume <100 µL. Phages were recovered by inverting the Amicon filter into a capless microcentrifuge tube and centrifuging at 1000× *g* for 2 min at room temperature. All collected phage samples (wash and elution steps) were stored at 4 °C for titering and/or amplification.

Eluted phages (~100 µL) were amplified in 25 mL NZY/20 µg/mL tetracycline medium following infection into starved K91BluKan *E. coli* host cells (~200 µL). Aliquots of each sublibrary was collected after 45 min of incubation and plated on NZY/Tet agar plates for quantification of infected colonies. Phages were purified and concentrated from the overnight culture using standardized double PEG/NaCl precipitation procedures. Phage sublibraries generated following each depletion strategy were maintained independently. Phages recovered in each wash step were titered by preparing serial dilutions of each sample in 1X TBS, pH 7.5 followed by infection of a 10 µL aliquot into an equal volume of K91BluKan *E. coli* starved cells for 15 min at room temperature. Infected *E. coli* cells were incubated with 180 µL NZY broth containing 0.2 µg/mL tetracycline at 37 °C for 45 min for induction of the tetracycline resistance genes found in the phage genome. Samples were then plated on NZY/Tet agar plates and incubated at 37 °C overnight.

(C) Subsequent Rounds of Selection—For each additional round of selection, an aliquot containing ~2.0 × 10^11^ virions of the amplified phage sublibrary from the preceding round was prepared in library dilution buffer (1X TBS, pH 7.5/0.5% Tween 20/0.01% BSA). As above, 50 µL of diluted phage sublibrary was incubated in 8 wells with adsorbed CCN1 protein without any additional depletion steps. For all rounds of selection, the concentration of CCN1 incubated in each of the wells was maintained at 10 µg/mL (500 ng/well) as in the first round of selection. In a duplicated fourth round of selection with the BSA-depleted library, the concentration of CCN1 was reduced to 1 µg/mL (50 ng/well) to enrich the library for phages with higher affinity towards the bound CCN1 protein. All washing, elution, titering, and library amplification procedures were performed as indicated in Section B above.

### 2.3. Screening Candidate CCN1-Binding Phages by Phage ELISA

**Figure 2:**
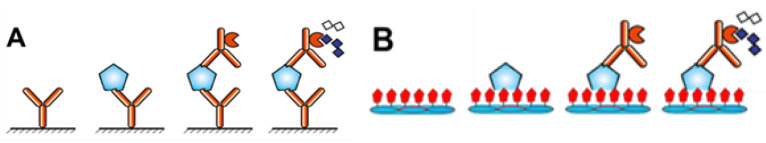
Sandwich CCN1 ELISA vs. Phage-CCN1 ELISA. (**A**) An anti-CCN1 antibody or (**B**) CCN1-specific phage bioprobe is immobilized onto ELISA plates. Soluble CCN1 protein analytes, or biofluids, are incubated until equilibrium is reached. An enzyme-linked detection antibody is added in the presence of a substrate to catalyze the appearance of a colored or fluorescent product and measured using an ELISA plate reader with appropriate detectors.

Individual phage clones identified following bioinformatics analysis were propagated and purified as described above. Phage clones were screened for binding to recombinant CCN1 protein using a phage ELISA, as illustrated in Figure 2.

Briefly, each unique clone was diluted to a final concentration of ~5.0 × 10^11^ virions/mL in 1X Coating Buffer (1X TBS, pH 7.5). Fifty microliters of each diluted phage was added to the wells of a high-bind, 96-well microplate (#9018; Corning, Corning, NY, USA) and adsorbed to the plate at 4 °C overnight. Phage solutions were removed, and wells washed three times with 200 µL 1X TBS, pH 7.5 to remove unbound phages. Wells were blocked with 200 µL Blocking Buffer (1X TBS, pH 7.5/0.1% Tween 20/1% BSA) for 1 h at room temperature. Solutions were removed and wells washed five times with 200 µL Wash Buffer (1X TBS, pH 7.5/0.1% Tween 20). Recombinant human CCN1 protein (#cyt-164; Prospec, Ness Ziona, Israel) was diluted to a final concentration of 1 µg/mL in Blocking Buffer and 100 µL (100 ng) was applied to each well. In control samples, 100 µL Blocking Buffer was applied to each well. Samples were incubated at room temperature for 1 h to reach equilibrium. Unbound protein was removed and wells washed five times with 200 µL Wash Buffer. Wells were treated with 100 µL of a 1:2000 mouse anti-Cyr61/CCN1 monoclonal IgG (clone H-2 [RRID: AB_10608730]; Santa Cruz Biotechnology, Dallas, TX, USA) diluted in Blocking Buffer at room temperature for 1 h. Unbound antibody was removed, and wells washed five times with 200 µL Wash Buffer. Wells were treated with 100 µL of a 1:5000 horseradish peroxidase-conjugated, AffiniPure rabbit anti-mouse IgG (H + L) secondary antibody (#315-035-003 [RRID: AB_2340061]; Jackson ImmunoResearch, West Grove, PA, USA) diluted in Blocking Buffer at room temperature for 1 h. Unbound antibody was removed, and wells washed five times with 200 µL Wash Buffer. Wells were treated with 50 µL 1-Step Ultra TMB Substrate Solution (#34028; Thermo Scientific, Rockford, IL, USA) at room temperature for 5 min. Color development was stopped by addition of 50 µL Stop Solution (0.2 M sulfuric acid). Optical density of each sample was quantified at 450 nm using a Synergy H1 plate reader (Agilent, Santa Clara, CA, USA) and the pathlength corrected at 975 nm with a reference wavelength of 900 nm using the BioTek Gen5 software (version 1.11.5, Agilent, Santa Clara, CA, USA).

Results are presented as the mean sample absorbance ± sample standard deviation of duplicate measurements. A Student’s *t*-test was performed on each phage clone to compare the mean optical density of replicate samples incubated with CCN1 protein compared to treatment without CCN1 protein (only BSA). Clones indicating statistically significant differences in optical density were identified with a *p*-value ≤ 0.05. A specificity ratio of selected phages was calculated by dividing the mean OD_450_ of CCN1 treated samples with the mean OD_450_ of BSA treated samples. The standard deviation of each specificity ratio was calculated as follows:

(1) $$\sigma =\sqrt{\frac{\;\sum {\left(\sigma_{r}\right)}^{2}}{\left(n-1\right)}}$$

where σ_r_ is the standard deviation of each ratio and n is the number of ratios. Clones with a specificity ratio greater than 2.0 were considered positive for capturing soluble CCN1 from solution and produced a signal significantly higher than the background.

### 2.4. Molecular Modeling

To develop structural protein models of phage displayed peptides on the filamentous bacteriophage fd-tet we used homology modeling with Modeller (v 10.6) [37]. Briefly, the cryo-EM structure of the filamentous bacteriophage fd major coat protein complex (PDB ID: 8CH5) was used as template for construction of new protein models [38]. Fifty copies of the full-length primary protein sequence of identified landscape phage displayed sequences were aligned to the template sequence. The positions of missing N-terminal residues of each major coat protein were generated using the DOPELoopModel class on the first 15 residues (AXXXXXXXXXPAKAA, where X is any amino acid residue) followed by a fast molecular dynamics refinement to minimize structure energies. Quality of loop refinement for each generated model was assessed by normalized DOPE scoring. Models generating the lowest score were selected for further refinement/molecular docking. To develop a structural protein model of the mature CCN1/Cyr61, the human CCN1 primary protein sequence, excluding a 24 bp signal peptide, was obtained from the NCBI RefSeq database (accession NP_001545.2) and submitted to the Google AlphaFold3 Server using default settings for construction of a model [39]. PDB structures were cleaned of ions and mislabeled atoms before completing 50,000 steps of steepest descent energy minimization (0.01 nm/step) for each individual structure with GROMACS (v. 2023.1) using the AMBER99 protein force field and a physiological concentration of Na^+^ and Cl^−^ ions until the minimization converged to a maximum force <10 kJ mol^−1^ nm^−1^.

To perform protein-protein molecular docking we used HADDOCK web server (v. 2.5-2024.12) [40,41,42] on structural models of fd phage major coat protein complexes displaying recombinant fusion peptides and the mature CCN1. Energy minimized PDB structures were uploaded to the HADDOCK web interface and active binding residues were defined. Four molecular docking simulations were completed using default settings and a hundred amino acid, non-overlapping sliding window region defined on the CCN1 pdb model to contain the active binding residues. Docking clusters were sorted by HADDOCK score, reflecting an estimate for overall binding energy, and Z-score, reflecting the standard deviation of the cluster. Selected clusters were submitted to the PROtein binDIng enerGY (PRODIGY) web service for prediction of binding affinity and identification of interacting contacts [43].

## 3. Results

### 3.1. Isolation of CCN1-Binding Landscape Phages

**Figure 3:**
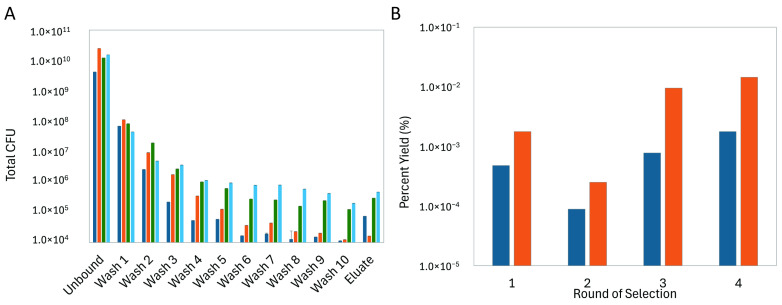
Selection of CCN1-binding phages. (**A**) Representative titering data of phages recovered from sequential selection steps. Samples were collected from each fraction (unbound, washes, or eluted) and titered in *E. coli* host cells. Bar plots show the mean ± standard deviation of the calculated total CFU present in each fraction by round. Bars are colored by the round of selection: Round 1 (dark blue), Round 2 (orange), Round 3 (green), and Round 4 (light blue) (**B**) Library enrichment, expressed as the percent yield (% yield = eluted output phage in CFU/input phage in CFU × 100) by round and depletion strategy. Recovered phages from each round of selection were isolated and amplified in *E. coli* host cells. Comparison of library enrichment between two different depletion strategies: depletion against plastic followed by adsorbed BSA (blue), or depletion against adsorbed BSA (orange).

To discover novel phage proteins that interact with purified CCN1 protein, we screened a multibillion clone f8/9 phage display library that has been thoroughly examined to identify peptide ligands against various targets of interest to biomedical researchers including isolated proteins [24], bacterial cells [28], mammalian cells [44,45,46,47,48], tumor xenografts, and whole animal tissue navigation [49]. Here, we enriched a population of phages through 4 rounds of selection against recombinant hCCN1 protein adsorbed to the wells of a 96-well microplate, as depicted schematically in Figure 1B. We performed a selection procedure previously used to identify phages binding different forms of prostate specific antigen (PSA) [26]. Peptide diversities of phage display libraries can be significantly impacted by enrichment of fast-growing, non-specific and/or plastic-binding peptides in the first round of selection that out compete phages displaying an antigen-specific peptide sequence [50,51,52]. Here, to reduce the number of non-specific and plastic-binding phage clones, we compared the effect of library composition between two different depletion strategies: (1) depletion against plastic followed by depletion against BSA, and (2) depletion against BSA. We observed a 1000-fold decrease in titered phages recovered from the unbound fraction prior to the first round of selection when a plastic depletion step was included in the procedure. This suggests that a significant portion of the library was lost during the plastic depletion step; resulting in a loss of population diversity in the initial library. We followed the enrichment of the two depleted phage libraries towards CCN1 protein using standard procedures. After the first round of selection, we observed a large increase in recovered phages as indicated by the percent yield for each depletion strategy. We observed an ~10-fold increase in eluted phages after each successive round of selection indicating successful enrichment of phage clones within the population (Figure 3). Fractions of each subsequent stage of the selection process were titered in *E. coli* cells to quantify the total number of phages in each step. As expected, we saw a significant decrease in total number of phages recovered in washes as the washing steps proceeded (Figure 3A). As we proceeded through each round of selection, the total number of phages recovered in each fraction increased suggesting a general growth in phage affinity towards the CCN1 protein and more phages were being eluted off during sample equilibrium.

We suspect that during the first round of selection, a highly diverse population of CCN1-binding phages were captured and amplified, but only those with strong binding were recovered in the second round which caused a significant drop in the percent yield (Figure 3B). A random sampling of clones was screened at the completion of the second round to verify an essential diversity of clones was still available to proceed through the selection procedure.

During the fourth round of selection with the BSA-depleted library, we tested the effect of adsorbed CCN1 protein concentration to further enrich the phage population for higher affinity clones. Based on the yield following each round of selection, the overall yield was significantly higher when 10 µg CCN1 protein was adsorbed to each of the wells as compared to wells treated with 1 µg CCN1 protein. As expected, more total clones were recovered when using a higher mass of CCN1 protein in each of the wells. However, it is expected that when less protein is used, clones that remain bound to CCN1 will demonstrate increased specificity or affinity towards the target protein.

### 3.2. Discovery and Analysis of CCN1-Binding Phages

At the completion of the final round of selection, one hundred phage clones were sequenced to identify the most prominent clones enriched during the selection process and identify peptide families representing peptide motifs. Overall, 26 structurally unique peptide sequences (Table 1) were identified across all final rounds of selection with phages displaying one of the three most prominent peptides on the N-terminus of the mature p8 protein: GSESVDMPA (26/69 = 37.7%), AFVYDDAAD (11/69 = 15.9%), or DIVYFDNSD (7/69 = 10.1%). In general, most identified clones were uniquely isolated from their respective round of selection. One phage clone could be identified across all rounds of selection (GSESVDMPA). While only five phage clones could be found in multiple rounds of selection, with most of the shared phage clones being recovered at the conclusion of the fourth round (AFVYDDAAD, ASDSDAFSG, and GSESVDMPA).

We performed a manual clustering of phages into families based on the identification of trimer consensus motifs found within each of the displayed peptide sequences (Table 2). Several families were enriched for serine and acidic residues (aspartic acid and glutamic acid) suggesting increased hydrogen bonding and electrostatic interactions between phages and isolated CCN1 protein. Enrichment of phage libraries with highly hydrophobic peptide sequences can indicate isolation of plastic binding motifs [53]. When comparing families identified in round 3 following the different depletion methods, we observed no common motifs between either sample suggesting the initial depletion step against plastic may have significantly altered the repertoire of phages available to bind the target protein. Analyzing motifs following the single depletion step against BSA revealed a commonly isolated RGD trimer motif which is involved in binding to various integrins. The binding specificity may further be regulated by the amino acid residue following the RGD trimer motif [54,55]. CCN1 interacts with extracellular integrins to regulate various biological processes and contains an integrin α_5_β_3_ (alpha 5-beta 3) binding site in domain II [56,57] and an α_6_β_1_ (alpha 6-beta 1) binding site in domain III [58]. Discovery of the RGD motif suggests that the isolated phage clone may interact in similar regions but would require further study to validate.

We similarly compared the diversity of families discovered following a fourth round of selection and hypothesized that a lower concentration of CCN1 protein would enhance the enrichment of lower binding affinity motifs. Overall, we observed no significant change in population diversity or change in proportions of specific amino acid residues between 10 µg and 1 µg CCN1 protein. Increasing the selection stringency for enhanced binding to CCN1 in round 4 resulted in an increased length of the motif from DXXSXFPD in round 3 to [D/S]XXS[D/E]FPD in round 4 and resulted in increased diversity of clones identified in the GS[D/E]SV[D/E]MPA family. It remains to be seen if the identified family would have an increased affinity towards CCN1.

### 3.3. Specificity of CCN1-Binding Phage Clones

**Figure 4:**
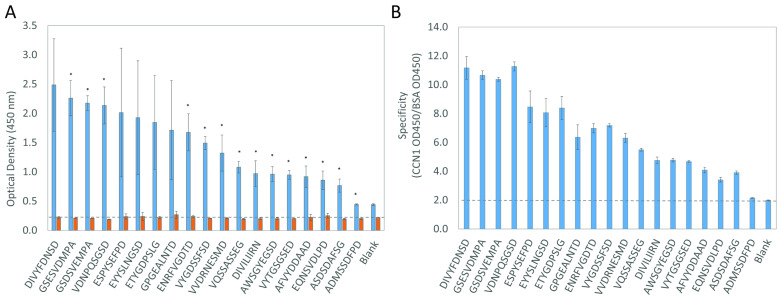
Specificity of CCN1-binding by Phage ELISA. (**A**) Phage ELISA comparing the ability of a phage clone to capture soluble CCN1 protein (blue) compared to BSA (orange). (**B**) Phage specificity ratio defines the ability of an immobilized phage clone to discriminate CCN1 binding from background signal. Bar charts display the mean ± standard deviation of technical replicates for each phage [N = 2]. A dashed line represents the background threshold set from a blank sample. Statistically significant differences (*p*-value ≤ 0.05) as calculated by a Student’s *t*-test are indicated by a star.

To determine the relative binding capacity of phages to CCN1 protein and determine the capability of phages to capture soluble CCN1 from a liquid medium, as would be used in a diagnostic liquid biopsy, we performed a phage ELISA to screen candidate phage clones. Here, we adsorbed a panel of candidate phages into the wells of a 96-well microplate and incubated each isolated phage with a solution containing a known concentration of CCN1. Bound CCN1 was then quantified by indirect ELISA using a commercial anti-CCN1 antibody [59]. We compared the binding of each phage towards CCN1 to the binding towards BSA, an unrelated carrier protein used in ELISA blocking and washing buffers. We defined the specificity of each phage clone as the ability of the fusion peptide displayed across the surface of the phage to interact with the target protein (CCN1) in comparison with an unrelated protein target (BSA). Nineteen different phage clones were screened as representative candidates following our bioinformatics analysis (Figure 4).

Each identified phage had increased Phage ELISA signals compared to an unrelated BSA protein (Figure 4A). We saw a slight increase in background signal for the blank and one phage sample which indicates that further optimization of antibody concentrations is needed for further assays. However, we identified phage clones displaying similar peptide sequences (i.e., GSESVDMPA and GSDSVEMPA) clustered together and had similar binding affinities. Similarly, we observed that each clone had a significantly higher specificity towards CCN1 than BSA when compared to a blank solution (Figure 4B). Together these data demonstrate that the phage clones identified through the selection procedure were enriched for peptides binding immobilized CCN1 protein, as illustrated by phage capture assay (Figure 1B). To test whether phages enriched in round 4 with a lower concentration of CCN1 would produce phages with higher affinity, we ranked each clone by specificity ratio and compared their relative abundance from each round of selection. There was no clear trend to demonstrate that phages selected with 1 µg of protein compared to 10 µg of protein enriched the population for higher affinity binding sequences (Table 1). Several peptide sequences identified from the selection experiment with lower protein input (i.e., GSDVEMPA, ESPYSEFPD, and ENRFVGDTD) demonstrated higher binding and were not very abundant in previous rounds of selection. Phage clones with lower binding identified in round 4 when using higher protein concentrations (i.e., VVDRNESMD, VQSSASSEG, and DIVILIIRN) were not found in the phage sublibrary from round 4 with a lower protein input. However, clones like AFVYDDAAD, which were more abundant in round 4 when a higher protein input was used, were significantly reduced in abundance. Reducing the concentration of immobilized protein might reduce the abundance of rapidly growing phages within a population and allow enrichment of additional binding sequences.

### 3.4. Identification of Putative CCN1-Binding Sites by Molecular Docking

**Figure 5:**
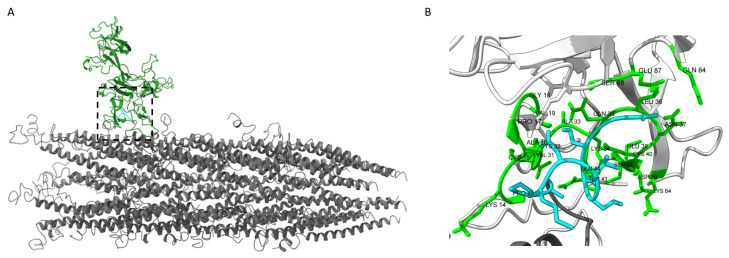
Molecular model of CCN1 bound to recombinant bacteriophage bioprobes. (**A**) Segment (~20 nm) of filamentous bacteriophage GSESVDMPA containing 50 copies of fusion p8 major coat proteins (gray). Predicted binding site of CCN1 protein (green) bound with the GSESVDMPA peptide fusion (light blue) displayed at the N-terminus of the p8 major coat protein. (**B**) Amino acid residues mediating contacts between CCN1 protein (green) and the phage-displayed GSESVDMPA fusion peptide (light blue).

We next sought to determine a potential model for phage binding with recombinant CCN1 protein using homology modeling and protein-protein docking methods (Figure 5). Well defined molecular structures of the p8 major coat protein from filamentous bacteriophage fd/M13 have been solved and subsequently studied by several groups using different techniques including solid-state NMR [60,61] and cryo-EM [38,62,63]. The p8 coat protein N-terminal domain is often unresolved in molecular models because of highly disorganized and flexible structures. As a recombinant insert displayed on the N-terminus of p8 coat proteins would remain largely unresolved using current structural biology methods [64], we proposed construction of a model based on homology modeling [65]. We used Modeller to construct a structural protein model of the p8 coat protein with the N-terminal domain displaying the most predominant peptide identified from our study, GSESVDMPA. Resulting models produced structures with multiple confirmations of N-terminal domains across the surface of the phage. These models demonstrate the diversity of conformations displayed across each landscape phage clone which result in the generation of structurally unique landscapes across the surface of each particle based on the properties of the displayed fusion peptide.

Molecular docking of the recombinant phage model with the mature CCN1 protein structure resulted in 39 different clusters of docked structures. Interacting contacts between CCN1 and the fusion peptide from the top 2 structure clusters were identified using the PRODIGY webserver to predict residues involved in binding. Binding contacts with GSESVDMPA peptide were predicted in the N-terminal Insulin-like Growth Factor Binding Protein (IGFBP) domain of CCN1, with some additional supporting interactions found in the Von Willebrand Factor Type C domain (VWFC) as visualized in Figure 5B. Some models predicted binding in the C-terminal Cystine Knot-like domain. However, the results of the phage ELISA, which uses an anti-CCN1 monoclonal antibody (clone H2), in combination with molecular modeling do not support this model due to spatial constraints imposed by the binding antibody. The binding affinity and dissociation constant between the docked CCN1 protein with the phage bioprobes under physiological conditions are estimated at ΔG = −10.4 kcal/mol and K_D_ = ~4.5 × 10^−8^ M, respectively. Additional experimental validation is needed to validate the proposed structural model. However, development of a protein model for CCN1-phage binding allows us to generate hypotheses about the binding specificity towards other proteins containing IGFBP or VWFC domains.

## 4. Discussion and Conclusions

CCN1 has been implicated as an important regulator of cell proliferation and serves as a mediator of metastatic disease progression. Development of therapeutic interventions that target CCN1 with monoclonal antibodies binding the VWC domain, as identified through epitope mapping with phage display, have been used to inhibit cell growth and prevent metastases in MDA-MB-231 breast cancer cells in vitro and in vivo [66]. Here, we demonstrate the ability of a landscape phage display library to contain short 9-mer peptides that interact with CCN1, a multi-domain, eukaryotic globular protein with an average molecular weight of ~42 kDa that serves as a model for development of additional peptide probes towards other soluble extracellular proteins [67]. Using an immobilized recombinant protein, the phage library was subject to 4 rounds of enrichment to yield a population of phage clones displaying peptides with specificity towards the captured target protein. We tested two depletion strategies to reduce the number of non-target binding phage clones from the enrichment; a common issue in biopanning experiments where fast-growing, low-affinity clones may dominate the population of clones if not removed from the experiment at an early round of enrichment. We observed a rapid loss of phages when using a plastic depletion step which is assumed to be due to the highly engineered surface treatment of the plastic microwells to capture proteins with diverse sequences. Phages that were not captured during the plastic depletion step may represent sequences difficult to bind to the treated microwell plates and can be used to further identify proteins that are challenging to adsorb to various treated-plastic consumables used in diagnostic assays like ELISAs. Since the plastic depletion steps significantly reduced the total population of available phages, we chose to further enrich the phage populations using a non-plastic depletion method in favor of depletion against only the blocking reagent, BSA.

Irrespective of depletion method, we were successfully able to identify several peptide sequences that interact specifically with the immobilized CCN1 protein and not an unrelated target. We screened a panel of these peptide sequences in a phage ELISA, an assay format that could be further used for development of diagnostic assays, and demonstrated that the binding for several of the identified sequences clustered into similar families. We conclude that these families of peptides are likely binding to similar binding sites located on the CCN1 protein. Additional modeling and experimental assays would be necessary to identify the specific binding site of each phage clone. One limitation of our study is the use of a non-glycosylated form of CCN1 protein during selection. CCN1 contains several glycosylation sites and post translational modifications that serve in critical roles for its secretion [68,69] and its ability to interact with other downstream molecules [70]. Identification of peptides binding to the non-glycosylated form of CCN1 allows for development of a diagnostic assay containing multiple peptide sequences to monitor glycosylation status at site-specific locations within the mature CCN1 molecule. Identification and validation of these peptide sequences with glycosylated forms would still be required. In this study, we proposed using homology modeling and molecular docking to define the amino acid residues involved in binding between the selected 9-mer peptide fusion peptides and the mature form of CCN1 protein. Further validation of these models using experimental binding assays with other members of the CCN family of proteins is necessary to confirm the binding sites and selectivity of the phage probes. Validation of computational methods to predict binding interactions discovered through biopanning experiments will ultimately assist in engineering novel proteins or rapidly screen identified peptide leads without labor-intensive in vitro experiments.

We demonstrate that phage-displayed 9-mer peptides can be identified from a landscape phage display library and those selected phages can be used as a stable capture probe for use in a diagnostic assay format, here a phage ELISA. Here we identified a panel of CCN1-binding phages that can be used to validate an ELISA-based diagnostic assay for the early detection of breast cancer from a liquid biopsy for quantification of soluble serum biomarkers.

## Figures and Tables

**Table 1:** Frequency of identified CCN1-binding phages by round.

Sequence *	Round 3 (Plastic and BSA)	Round 3 (BSA only)	Round 4 (10 µg CCN1)	Round 4 (1 µg CCN1)
ADMSSDFPD	-	1	-	1
**AFVYDDAAD**	-	-	9	2
ASDSDAFSG	-	-	1	3
AWSGYEGSD	1	-	-	-
DIGVMAENE	-	-	1	-
DIVILIIRN	-	-	1	-
**DIVYFDNSD**	-	-	7	-
DTTGSGVDG	-	1	-	-
EDDDSSFPD	-	1	-	-
**ENRFVGDTD**	-	-	-	1
EQNSVDLPD	1	-	-	-
**ESPYSEFPD**	2	-	-	1
**ETYGDPSLG**	-	-	1	-
**EYYSLNGSD**	1	-	-	-
**GPGEALNTD**	1	-	-	-
GQYEQSVAE	-	-	-	1
GSDSVDMPA	-	-	-	1
**GSDSVEMPA**	-	-	-	1
**GSESVDMPA**	10	7	2	7
**VDNPQSGSD**	1	-	-	-
VGRGDGNED	-	1	-	-
VQSSASSEG	-	-	1	-
**VVDRNESMD**	-	3	1	-
VVQDRSADD	1	-	-	-
**VYGDSSFSD**	-	1	-	-
VYTGSGSED	1	-	-	-

* Phage-displayed foreign peptides are sorted alphabetically in the first column. Highly prominent phage clones that demonstrate a high frequency of occurrence are highlighted in bold.

**Table 2:** Identification of phage families as potential CCN1-binding sites based on consensus motifs found in selected peptides.

Round 3 (Plastic & BSA Depletion)		
**GS[D/E]**	**SVD**	**SGS[D/E]**
AWSGYE**GSD**	EQN**SVD**LPD	VDNPQ**SGSD**
EYYSLN**GSD**	GSE**SVD**MPA	VYTG**SGSE**D
**GSE**SVDMPA		
VDNPQS**GSD**		
VYTGS**GSE**D		
**Round 3 (BSA Depletion)**		
**DXXSXFPD**	**RGD**	
A**D**MS**S**D**FPD**	VG**RGD**GNED	
E**D**DD**S**S**FPD**		
**Round 4 (10 µg CCN1)**		
**DIV**	**S[D/E]S**	
**DIV**ILIIRN	A**SDS**DAFSG	
**DIV**YFDNSD	G**SES**VDMPA	
**Round 4 (1 µg CCN1)**		
**S[D/E]FPD**	**GS[D/E]SV[D/E]MPA**	
ADMS**SDFPD**	**GSDSVDMPA**	
ESPY**SEFPD**	**GSDSVEMPA**	
	**GSESVDMPA**	

## Data Availability

Data that supports the findings of this study are available within the article. Any additional supporting data is available from the corresponding authors upon request.
